# Mobile Medical Education (MoMEd) - how mobile information resources contribute to learning for undergraduate clinical students - a mixed methods study

**DOI:** 10.1186/1472-6920-12-1

**Published:** 2012-01-12

**Authors:** Bethany S Davies, Jethin Rafique, Tim R Vincent, Jil Fairclough, Mark H Packer, Richard Vincent, Inam Haq

**Affiliations:** 1 Brighton and Sussex Medical School, Brighton, East Sussex, UK

## Abstract

**Background:**

Mobile technology is increasingly being used by clinicians to access up-to-date information for patient care. These offer learning opportunities in the clinical setting for medical students but the underlying pedagogic theories are not clear. A conceptual framework is needed to understand these further. Our initial questions were how the medical students used the technology, how it enabled them to learn and what theoretical underpinning supported the learning.

**Methods:**

387 medical students were provided with a personal digital assistant (PDA) loaded with medical resources for the duration of their clinical studies. Outcomes were assessed by a mixed-methods triangulation approach using qualitative and quantitative analysis of surveys, focus groups and usage tracking data.

**Results:**

Learning occurred in context with timely access to key facts and through consolidation of knowledge via repetition. The PDA was an important addition to the learning ecology rather than a replacement. Contextual factors impacted on use both positively and negatively. Barriers included concerns of interrupting the clinical interaction and of negative responses from teachers and patients. Students preferred a future involving smartphone platforms.

**Conclusions:**

This is the first study to describe the learning ecology and pedagogic basis behind the use of mobile learning technologies in a large cohort of undergraduate medical students in the clinical environment. We have developed a model for mobile learning in the clinical setting that shows how different theories contribute to its use taking into account positive and negative contextual factors.

The lessons from this study are transferable internationally, to other health care professions and to the development of similar initiatives with newer technology such as smartphones or tablet computers.

## Background

Medical students need to assimilate considerable new information during their studies especially with the need for evidence based practice, and they must develop skills for lifelong learning, keeping their knowledge updated [1]. Lifelong learning, particularly in medicine, requires motivation and problem identification and solving skills relevant to the clinical situation [2]. There have been rapid advances in the development of new teaching methods and learning resources, and a considerable enhancement in the availability of electronic and mobile resources. Recent news reports show an increasing use of mobile and smartphones by young age groups with easy access to the internet and medical 'apps'[3]. Handheld computers are widely used by clinicians during the delivery of care for accessing up-to-date medical references, especially drug formularies [4]. Their use in medical practice and education is in line with the General Medical Council's (GMC) requirements [1] and is generally thought to be of great benefit to both teachers and students[5]. Given their utility after qualification undergraduate medical students may benefit from earlier exposure, both to improve their skills and work habits with a mobile device, and by enhanced learning opportunities.

There are several studies assessing the practical use of mobile devices and handheld computers in the medical education setting, but what is lacking to date is the underpinning pedagogic basis for the use of such technology [6,7]. If limited resources are to be directed towards the provision of technology, it needs to be justifiable from a theoretical perspective and regarding outcomes of such an intervention. Research in e-learning to date has concentrated on demonstrating efficacy and comparing new technology with old [8]. What is needed to inform future use is further research directed towards 'when' and 'how' to use e-learning. Although this may be context specific, educators elsewhere can judge the transferability and relevance for their own institution.

There are several independent theories of learning which may be involved in mobile learning, but we do not know to what extent, or how they may interact with other contextual factors such as environment and prevailing culture [9]. A conceptual framework would help us understand how to maximize the effectiveness of mobile learning. The main questions that we intended to answer through our study were: how the medical students used the technology, how it enabled them to learn, what learning theories contributed, what barriers were encountered and what could be done to overcome them. We did this by providing high quality educational resources on a mobile device, and conducting qualitative and quantitative research using focus groups, questionnaires and tracking data.

## Methods

The study was an extension of a successful pilot study of 20 year 3 students in 2006/7 [10].

### Ethical approval

Ethical approval for the study was given by Brighton and Sussex Medical School Ethics Committee

### Participants

All students in years 3-5 of the medical school were invited to participate. The study was advertised at core student lectures, on the virtual learning environment and by emailed invitations to all students. There were 419 clinical students enrolled on the course when the study began. The medical school was newly established in 2003 and runs a 5 year integrated course for a mixture of school leavers and graduate entry students.

### Materials and costs

Participants were loaned a Hewlett Packard iPAQ 114 Classic handheld PDA with a suite of software on an SD memory card known as DrCompanion (supplied by MedHand International). The resources included the British National Formulary (BNF) and textbooks such as the Oxford Handbooks and Netter's Anatomy (Table 1). Although smartphones were available at this time, the cost of supplying all students with such a phone would have been prohibitive compared to supplying a PDA. Also, MedHand International had not yet developed a version suitable for common smartphone platforms at the start of the study.

**Table 1:** Resources provided on DrCompanion SD card

British National Formulary (BNF)
Chemical Laboratory References (CLAB)

Classification of Surgical Operations and Procedures (OPCS-4)

Clinical Evidence (CLEV)

Cochrane Abstracts (COAB)

DSM IV (EDSM)

EBM Guidelines (EBMG)

ICD 10 (EICD)

National Institute for Clinical Excellence (NICE)

Netter Atlas of Human Anatomy (NETT)

Oxford Concise Medical Dictionary (OCMD)

Oxford Handbook of Accident & Emergency Medicine (OHAE)

Oxford Handbook of Clinical & Laboratory Investigation (OHCL)

Oxford Handbook of Clinical Medicine (OHCM)

Oxford Handbook of Clinical Surgery (OHSU)

Oxford Handbook of Drug Therapy (OHDT)

Oxford Handbook of General Practice (OHGP)

Oxford Handbook of Medical Sciences (OHMS)

Patient Organizations (EPRG)

Although provided free of charge to the students themselves, the cost per student (inclusive of hardware and software) was approximately £520 over three years (~£170 per student per annum). There were additional overhead costs which ran at approximately £20,000 per year covering development, education, usage monitoring and content administration.

### Technical support

Introductory lectures enabled handout of the devices and training. Ongoing support was provided initially through a regular drop-in surgery and after this queries were handled through email or by a library IT technician. Other measures included a frequently asked questions (FAQ) page delivered through studentcentral (virtual learning environment based on Blackboard LearnM^®^ v9.1) as well as a screencast on how to update the DrCompanion cards.

### Design

The study was a prospective observational study

### Procedure

A triangulation approach was used to ensure the validity of the study with three main instruments used to assess the following questions of how learning is enabled by the use of mobile devices:

Surveys and tracking data for the practical aspects - where and when the devices were used, what resources were used the most, what prevented use, what encouraged use.

Focus groups were held to further explore these areas and also to establish how the mobile devices helped the students to learn, what was required of them and the establishment to make the most of the tool, what their personal experiences had been.

### Focus groups

Four focus group (FG) interview sessions (A-D) were held over the course of the academic year. A grounded theory approach was used with an iterative design and a constant comparison approach to data analysis. Convenience sampling was used to select participants as all students were invited by blanket invitation across the years by verbal and electronic advertisements. The questions were derived from the results of the pilot study in 2006/7 and the literature, and for the later focus groups, additional significant themes arising from earlier ones. The number of participants varied from 3 to 7 with each year group being represented. Each interview lasted approximately 45 minutes. The participants reflected a cross-section of the students involved in the study with regards to mobile device use and comfort with use of technology. Facilitators included both members of the study group and members of the University staff not involved with the study.

Audiotape recordings were made and transcribed verbatim by external staff. Students from each focus group confirmed that the transcripts were an authentic record of events. The transcriptions were checked for accuracy then analysed and coded independently by two researchers (BD, IH). BD had not been involved in the early development of the project, bringing further openness to the analysis. These were then organised into emergent themes, with careful attention paid to divergent views. Focus groups and analysis continued until no new themes emerged and data saturation was felt to be complete.

### Surveys

There were no suitable validated questionnaires available on review of the literature. Questionnaires in the 2006/7 pilot study [10]were developed from themes arising from the literature [11-14] and amended for use in a final form in this study.

Pre-study: Students were surveyed when given their PDA as to their current information technology (IT) skills and use of IT for educational purposes, their perceptions of the advantages and disadvantages of using a PDA and which resources they expected to find most useful.

Post-study: A 12-point questionnaire was devised and distributed to the 3^rd^ and 4^th^ year medical students at the end of their 1^st^ and 2^nd^ year of use respectively (July 2010). These were distributed at mandatory attendance events at which all students were expected to be present to increase yield, and collected at the end of the same event. The year 5 students were not surveyed as they had completed their studies. The items were developed to answer the research questions posed above and to assess the generalisability of some of the issues raised through the focus groups. The questions comprised a combination of structured and free response items. Questions 1 and 2 asked all respondents about their use of PDAs and how it compared to their expectations. Questions 3-7 on frequency and details of PDA and DrCompanion resource use, location of PDA use and examples of feedback from patients/teachers/hospital staff were answered only by the group stating they were using the PDAs. Questions 8 and 9 covering factors preventing PDA use and factors that would encourage their use were answered only by the group stating that they did not use their PDAs. Questions 10-12, answered by all respondents, asked whether they would like to return their PDA to the School in addition to ascertaining numbers of students with smartphones and feedback on future directions for the project. Questions 4-6 and 8-9 allowed multiple responses.

### Usage monitoring

Students were required to regularly synchronise their device to receive software updates. Upon synchronisation information was recorded on the resources used and number of pages viewed only. Synchronisation could not be done remotely and relied on each student to actively synchronise with a networked personal computer. Regular emails were sent to remind the students of the necessity for this. Useage monitoring was not available at the time of the pilot project [10].

## Results

419 students were enrolled in years 3-5 and 387 chose to participate in the study.

### Pre-study survey

302/387 (78%) responded, all of whom had a personal computer and 150/387(38%) already owned a handheld mobile device. The majority(68%) felt confident in their general IT skills and with the use of a mobile device. Initial perceptions of the advantages to using a PDA in medical education were the benefits of instant access and portability of the device. Disadvantages were thought to be loss or theft of the device, the development of dependency upon it and concerns that it might appear disrespectful.

### Post study survey (see Table 2)

**Table 2:** Results of post-study survey.

		Number	%
**1. Ever used PDA since distribution and teaching session on how to use it ?**	Yes	102	76
	
	No	32	24
**(N = 134)**			

**2. Usage now compared to student expectations**	Less	91	68
	
	Same	27	20
	
**(N = 134)**	More	13	10
	
	Far more than expected	3	2

**3. Frequency of use**	Hardly ever	35	34
	
**(N = 102)**	Once a month	19	19
	
	Once a week	35	34
	
	Daily	13	13

**4. Uses of PDA**	DrCompanion	101	99
	
**(N = 102)**	Other online medical resources	4	0.4
	
	Notes from learning experiences	6	6
	
	Email/calendar/diary	9	9
	
	Leisure (music, games)	17	17

**5. Most used DrCompanion**	OHCM	89	87
	
**resources (top four listed)**	BNF	91	89
	
**(N = 102)**	Medical dictionary	22	22
	
	Netter's anatomy	19	19

**6. Location of most common use of PDA?**	Home	28	27
	
	Library	0	0
	
**(N = 102)**	Within the trust	49	48
	
	In teaching	0	0
	
	On the move	10	10

**7. Feedback from**	Positive	16	16
	
**patients/teachers/others on use of PDA**	Negative	18	18
	
	Mixed positive and negative	7	7
	
**(N = 102)**	Neutral	15	15
	
	No response	46	45

**8. Factors preventing PDA use**	Carry another device	25	78
	
**(N = 32)**	Electronic device not preferred learning modality	13	41
	
	IT issues	6	19
	
	Confidence	5	16
	
	Theft/loss	9	28
	
	Other	5	16

**9. Factors that would encourage PDA use**	Training - PDA	3	9
	
	Training - DrCompanion	4	13
	
**(N = 32)**	Student demonstration	3	9
	
	Repair	3	9
	
	Insurance	6	19
	
	No response	9	28

**10. Student wishes to return PDA**	Yes	12	9
	
	No	36	27
**(N = 134)**			

	No response	86	64

**11. Number of students with smartphones (N = 134)**	Yes	49	37
	
	No	83	63

**12. Future of MoMEd project**	School does not provide students with any mobile learning devices nor resources to accompany them	3	2
	
**(N = 134)**	School continues to provide a basic PDA and DrCompanion resources	39	29
	
	Students use their own smartphone and School provides resources compatible with these phones	47	35
	
	School provides students with a smartphone and DrCompanion resources for the duration of the course	45	34

140 students responded (74/133 year 3 students, 66/123 year 4 students) with a response rate of 54.7% Year 5 students were not surveyed as they had already graduated but if included for "intention to treat" purposes, the response rate of the cohort as a whole was 140/387 (36%). Six responses were incomplete and 134 questionnaires were analysed. Of the 102 respondents that did use their PDA, 47% did so at least once a week, mostly within the clinical setting (48%) or at home (27%). In PDA users, the British National Formulary (89%) and Oxford Handbook of Clinical Medicine (87%) were the most popular resources. Feedback from patients and teachers was mixed but 45% students did not respond to this question. 32 respondents (24%) had not used their PDA, and in this group, the main reasons were cited as needing to carry another device (78%) and learning preferences (41%) and concerns around theft and loss (28%).

37% of all respondents had smartphones and 98% of all respondents wanted the initiative to continue either with the School providing DrCompanion resources with or without a PDA or smartphone

### Usage data

124/387 students enabled data logging by synchronising their devices (55 year 3, 41 year 4, 28 year 5 students). The students accessed the resources on their DrCompanion cards on average 68.5 times (median) over the monitored 10 month period (interquartile range 17.8 - 160.5). The most popular resources were the British National Formulary (BNF, a drug reference) and the Oxford Handbook of Clinical Medicine (OHCM) (Table 1).

### Qualitative analysis

The themes that emerged from the data analysis of the focus groups and free text responses within the surveys allowed us to answer our research questions.

### How students used the tool

The focus groups participants agreed with the survey results on the practical aspects of using the technology. They tended to use it mostly between patients or scheduled teaching activities, and less commonly during delivered teaching sessions. As was expected through the very nature of the mobile technology, using it 'on the go' was a recurrent statement, including locally and on elective (i.e. on clinical attachment abroad). For the most part they used to it to access quick references, with the most popular resources being the BNF and the OHCM, validating the questionnaire results, and shown by student quotes below:

• "I used it principally for making reference on the wards and I use the anatomy quite a lot when we are in theatres." (FG:C)

• "I tend to use the PDAs as a quick reference on the wards - for drugs and that sort of thing." (FG:C)

Some students continued to make use of the PDA at home even though similar resources could be accessed via a home computer. This highlighted the role of the mobile device as an additional tool rather than a replacement.

### How student learning was enabled

Four ways in which learning was enabled emerged from the focus group analysis. These were

1. Timely access to key facts -learning in context

2. Consolidation of knowledge through repetition

3. A supplement rather than a replacement

4. Making use of wasted time

Timely access to facts - learning in context:

The focus group participants described using the PDA/DrCompanion to learn whilst actively engaged in clinical activities as well as in spare moments, allowing them to learn in context:

• "When you see the patient and can access the information at the same time." (FG:B)

• "I' d never use it if I was actively talking to a patient. But again as soon as that conversation has finished I'm happy even if they're still around." (FG:B)

Consolidation of knowledge through repetition:

Students found that instant access through the mobile technology allowed them to repeatedly look up information with ease, reinforcing their knowledge. They recognised that this was an important part of learning and appreciated the opportunity offered:

• "Reinforcing key points at point of need." (FG:A)

• "Initially, you may look at it three times and then after that you will become more confident." (FG:A)

A supplement rather than a replacement:

Those students who had integrated the PDA/DrCompanion into their learning strategies recognised that its role lay most successfully as a supplement rather than a replacement:

• "I think it's complementary rather than substitution." (FG:C)

• "It is actually nice that it's there, because you know - it is handy, it's just another tool that you can use." (FG:D)

Making use of wasted time:

Another benefit was the ability to make the most of empty time spaces as accessing information via the PDA in spare moments was seen as an opportunity to make constructive use of time. These were short segments of time between formal scheduled events, or for example between patients in clinic. The portability of the PDA enabled them to spend time they felt otherwise wasted, learning:

• "You're absolutely right - that's a real plus. 'Carpe diem' - making use of time. Very much. I agree definitely." (FG:B)

• "Actually, that's one of the reasons I have started to use it a lot more. There and then when there isn't anything to do you can make use of time." (FG:B)

### Barriers that inhibited the learning opportunity

Careful analysis of the data showed that in addition to technological issues, there were more self-imposed and subtle barriers that had restricted the students. Many of them felt that using the PDA whilst in a clinical context interrupted the ongoing experience:

• "I tend to look for opportunities to use it when I'm not doing really anything else. Rather than using it and perhaps disrupting what else is going on." (FG:B)

• "Personally I prefer to kind of engage with the clinical situation then go away and read it as a separate thing." (FG:D)

There was also a widely expressed view that students had had negative experiences with patients and staff. Although this was hearsay for the most part rather than directly experienced, these negative perceptions left the students reluctant to openly use the technology on many occasions:

• "I think some people mentioned that if they were on the ward, some of the doctors thought they were using their phones." (FG:C)

• "I think some doctors have made comments about "What are you doing on that, are you texting someone, or playing games." (FG:D)

Students were concerned about having to carry another device, the possibility of theft, loss or damage and the electronic nature of the device.

Dislike of technology:

• "It's just that I've never been very techie." (FG:D)

• "But I just wonder if there is actually something which is more intuitive - with less extra effort - it might be more useful...." (FG:C)

Practical issues:

• "One or two occasions when it would just freeze or stop, or just get frozen on loading, and that's probably how I started using it less and less." (FG:D)

Extra device to carry:

• "The only thing is, you don't have that many pockets - certainly I don't. So, I would have my wallet in one pocket, my phone in another - because you can't keep your phone at home - and I found it quite hard to carry it around with me all the time." (FG:C)

### How barriers were overcome - necessity for change

The responses from the students showed that these barriers could be overcome, but needed both individual and institutional input to optimise the opportunity. Offering the opportunity to all, rather than just those who were already mobile technology-friendly, engaged students who would have missed out otherwise.

• "I was quite averse to it at first - I was one of the haters..." "What changed your mind?""I think it's actually finding I did use the PDA and it did come in handy several times. It just makes life a bit easier." (FG:C)

A change in attitude, behaviour and approach was required for the PDA to become an optimal tool, and a failure to change resulted in non-use or non-acceptance of the device. This was required both of the students and of the clinicians. If teachers were enthusiastic and advocated their use on ward rounds and in clinics then students were more likely to be encouraged to make it part of their routine. They had to find a way of working with the PDA to get the most out of it.

• "I know quite a few people who have just left them in their bedroom and have never touched them - it's off all the time." (FG:A)

• "It's things like that [teacher advocacy] which encourage you, maybe I will bring it with me tomorrow and take it on the ward round with me." (FG:B)

The same applied to their interactions with patients. As discussed above, the etiquette of using a PDA whilst with patients was of concern, and the students had to learn how to incorporate it into their consultations without harming their relationship with the patient:

• "I guess the patients need to be informed of what you are doing and not feel as if you are being distracted by something else whilst you are talking to them." (FG:A)

Focus group participants also felt that integration into a Smartphone platform would remove some of the barriers that they had encountered.

• "Now, I'm thinking maybe I will get an iPhone actually, because it might be really useful to have everything on a similar PDA-type idea but merged with a phone. I'm definitely quite keen." (FG:C)

### Conceptual framework

**Figure 1:**
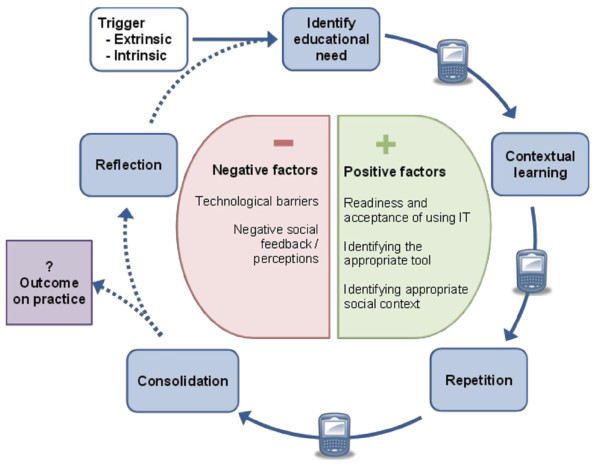
**Illustration of a model for mobile learning in the clinical setting showing influence of positive and negative contextual factors**.

We have developed a conceptual framework showing the contribution of current learning theories to mobile learning in the clinical setting and the impact of contextual factors, both positive and negative. Figure 1 illustrates a model based on our findings and it is discussed further in the next section. A trigger (external or internal) leads to the recognition of an educational need, following which the mobile device enables learning to take place. Positive and negative factors can affect the cycle at any stage. Broken arrows show areas where further research is needed.

## Discussion

This paper describes an innovative project (MoMEd) to evaluate mobile learning in clinical medical students. The conceptual model in Figure 1 shows the contribution of repetition and contextual learning theories to the process of mobile learning [15,16]. The development of abstract problem-solving schema requires the "repeated active application of the knowledge acquired" which is enabled by the PDA [17]. The instant access to information whilst in the clinical setting gives the students a better framework for understanding and storing the new information, and allows more efficient retrieval for future use [18]. The 'just in time' experiences related by our students is as described by 'reactive' or 'opportunistic' learning [19]. This is learning that is both intentional but "occurs in the middle of the action" rather than time being set aside for deliberate acquisition of knowledge, an apt description of the learning offered using the PDA.

Our model demonstrates how contextual factors can impact upon the learning process. Resources on the PDA were seen as a useful additional tool for them to have- a supplement rather than a replacement for their traditional learning strategies. This aligns with the theory of a learning ecology, an environment composed of a diverse variety of learning options, allowing each individual student to find the opportunity to access learning that addresses their own personal and immediate needs [20]. Appropriation in which users not only adjust the tool to best fit their activities, but the tool may also cause the user to change their behaviour to accommodate using the tool effectively, was also seen [21]. Home use of PDAs should also be seen as valid and part of the learning environment in mobile learning, with users able to access knowledge in different ways (PDA, book, personal computer) depending on the type and complexity of knowledge required. This finding also fits with the increasing popularity of tablet computers to access resources and the internet that are also available on home personal computers.

Both intrinsic mechanisms and external conditions may influence the learning possibilities available to a student [22]. As described in activity theory, the social context is important, with "social rules and conventions govern[ing] what is acceptable" and attitudes influenced by surrounding opinion [21]. Linked to this is the concept of acceptability of technology. The TAM model (Technology Acceptance Model) [23] suggested that usage of a system depended on several factors including perceived utility and ease of use that would determine the attitude towards technology and intention to use it. Holzinger [24] also suggested that previous exposure to technology and unobtrusiveness were also important factors that would affect acceptance and use of a technology. All respondents to our surveys had a personal computer and 38% already had another mobile device, suggesting that previous exposure was not a barrier to acceptance. The physical obtrusiveness of the PDA reflects the experiences of our students with respect to their concerns regarding teacher and patient opinion, and of a perceived failure to engage in the clinical moment, leading to potential inhibition of PDA use despite accepting its utility. This constrained their optimal use of the PDA. Alsos found that use of the device consumed the physician's attention, with poor action-transparency and inhibited patients from asking questions and raising issues [25]. Houston et al in contrast found that the majority of their patients had positive attitudes towards handheld computers, although the clinicians themselves had reservations [26].

With sufficient support these barriers can be overcome, but a need for change was paramount, from the student's own attitude and behaviours to that of the clinicians and patients. This was achieved through communication by the students of the authentic learning nature of the tool, and by encouragement by the clinicians. Guidance on etiquette was required, so-called "mobiquette" [5]. Institutional support helped both with encouragement and technical support, the importance of which has been highlighted by others [7,27]. Becoming a more formalised part of the curriculum may aid this.

There are some important learning theories that we were unable to demonstrate directly, but this does not mean that they do not contribute. Rather it is due to limitations of the research tools available. Experiential learning with reflection [28] is likely to have a key role as shown by the broken arrows in Figure 1, and further research to elicit this would be invaluable. We were also unable to demonstrate that learning that occurred affected the students' actual practice. Extending the methods of data capture along ethnographical lines may allow exploration of these but this is difficult given the very nature of mobile learning which is spread across many contexts and over long periods of time [29]. It makes it difficult to predict when a learning opportunity is likely to occur. Options include voice recordings or blogging, which may be more contemporaneous, or structured diaries have been used successfully [30]. An ethnographic approach would also allow assessment of the effect on student-teacher and student-patient interactions that a move to a smartphone platform may have, and to find how the students and patients find ways to manage this.

The strengths of this study are that a large cohort of UK medical students were engaged on whom both qualitative and quantitative data were collected on use of PDAs as learning tools in a clinical setting in the UK. Provision of the PDA and software enabled us to involve students beyond a small self selecting group keen on technology,so reducing bias.

There are limitations to the study. The surveys and focus groups were dependent on personal reporting and so subject to recall bias, and the quantitative tracking data was too limited to offset this. The focus groups may have had an inherent bias in that those who chose to participate had engaged more with the technology. Purposive sampling could have been used instead to reduce this. The survey response rates were lower than ideal leading to non-response bias, potentially limiting the generalisability of results. However triangulation of qualitative and quantitative results showed consistency and suggest the findings are valid. Information from the final year group would have been useful but were not surveyed as they were sitting their final examinations prior to graduation. The usage tracking developed for this study was limited in that it only showed the number of times a resource was accessed and relied on students synchronizing their PDA with a personal computer, which not all students did despite regular requests from the project team. Ideally, time of day, location and duration of resource use would have been useful data to collect, with automatic uploading of usage data through a wireless network, and this has now been developed for use in DrCompanion for smartphones. The project was pragmatic in that it gave the same device and resources to all students.

### Guiding future policy: research and resource design

Our research, although historical, has important lessons both globally as well as locally. PDAs are cheap and portable compared to standard computers, allowing use in rural and developing locations, and within the clinical setting. Although technology has advanced rapidly since this project began, the lessons from our research may be applied to settings in which the PDA may not be superseded for many years, and also to help to inform the emergent use of Smartphones. Smartphones may offer an advantage over classical e-learning as in many developing countries mobile telephone networks are a common alternative to networked computing [5]. A further argument for progressing down the smartphone route were the problems encountered with the now old and slow user interface and the necessity of carrying two devices. However the concerns regarding the acceptability of a mobile device by staff and patients may be exacerbated by using a smartphone platform, as a few students recognized. Ideally, the transfer from a PDA to smartphone approach could have been carried out earlier, however the institutional investment in buying and supplying PDAs to students, the length of the contract with MedHand International and the lack of availability of a reliable smartphone platform for DrCompanion until late 2010 led to a decision by the project team to continue with the approach described in this paper.

Arguments against the widespread introduction and integration of mobile technology within medical education include the lack of hard outcomes showing that learning is improved, and the expense of such a project. There are the obvious costs for the initial outlay for the devices and the licensing, but also the less easily recognised costs of providing technical support and repair costs. We have demonstrated that only a proportion of students make use of their device so it may be seen as a waste of resources providing them to all. An opt-in programme may be a feasible substitute but whatever strategy is employed, there needs to be equity among students. New software is now developed for smartphone use that allows additional information to be collected including time of access, duration and nature of viewed content to the level of page of particular resource and information will be sent automatically and directly to a secure server over a wireless internet connection. This will give useful information to investigate relationships between situational context (home, hospital, university)and resource use.

In addition to altering the delivery platform, the resources on offer to the students could be developed further. Integration with other aspects of the medical school's e-learning facilities could be considered, such as an online question bank and an e-portfolio. This has proved successful for the ALPS project already [31].

The resources supplied on the PDAs were versions of existing paper and online publications optimized as much as possible for a mobile platform. I n transferring or developing resources for future mobile platforms such as smartphones and tablets, design is important and relevant to medical education where use of the mobile resource occurs in environments where information is required quickly but also where access may be interrupted at short notice and continued at a later time and maybe a different location. Content also needs to be adjusted so that it can be easily viewed on a small screen (chunking) and minimizes the potential for the user to lose or give up on accessing information due to navigation issues. Resources also need to be able to work effectively on multiple hardware platforms that will have an impact on development costs [2,32].

## Conclusion

This is the first study to describe the learning ecology and pedagogic basis behind the use of mobile learning technologies in a large cohort of undergraduate medical students in the clinical environment. We have developed a model for mobile learning in the clinical setting that shows how different theories contribute and which will allow learning opportunities to be optimised.

The lessons from this study are transferable internationally, to other health care professions and to the development of similar initiatives with newer technology such as Smartphones or tablet computers. Further research on more defined long term outcomes of mobile learning is needed.

## Competing interests

The authors declare that they have no competing interests.

## Authors' contributions

BSD acquired and analysed data and drafted the manuscript. IH and RV conceived and designed the study. IH also contributed to analysing data and helped to draft the manuscript. MHP and TRV contributed to acquiring data. JF and JR acquired and analysed data. All authors read and approved the final manuscript.

## Pre-publication history

The pre-publication history for this paper can be accessed here:

http://www.biomedcentral.com/1472-6920/12/1/prepub
